# Effects of Transition from Closed-Book to Open-Book Assessment on Students’ Scores in a Pharmacokinetics Course

**DOI:** 10.3390/pharmacy11050134

**Published:** 2023-08-23

**Authors:** Reza Mehvar, Richard Beuttler

**Affiliations:** Department of Biomedical and Pharmaceutical Sciences, School of Pharmacy, Chapman University, Irvine, CA 92618, USA; rbeuttle@chapman.edu

**Keywords:** open-book assessment, closed-book assessment, remote assessment, in-person assessment, pharmacokinetics

## Abstract

Closed-book summative assessment of student learning, common in pharmacy education, is challenging to administer in a remote setting due to the need for costly and intrusive monitoring technology. Therefore, open-book assessments without monitoring have been considered an alternative in remote settings. The present study investigated the effects of the transition from in-person closed-book to remote open-book format on the students’ scores in different assessment categories in a Pharmacokinetics course. The students’ performances in the transition cohort (Transition, *n* = 96) during the in-person and remote periods were compared with those of an in-person cohort (Control, *n* = 85) during the same periods. Assessments included take-home assignments, daily quizzes, and progress/final examinations. Whereas the take-home assignments were open-book for cohorts and periods, the quizzes and examinations were open-book only for the Transition cohort during the remote period. Only the quiz/examination questions that were identical for both cohorts were included in the analysis. Statistical analysis by a linear, mixed-effects model indicated that the transition did not have any significant impact on the scores of students in the assignments, which were open-book for both cohorts and both periods. However, there were significant increases in the Transition cohort’s scores (mean ± SE) during the remote open-book period in both quizzes (+8.4 ± 1.9%) and examination (+6.8 ± 1.5%) questions, compared with the Control cohort who had in-person closed-book assessments. These differences amounted to Cohen’s *d*-effect sizes of 0.61 and 0.59 for the quiz and examination questions, respectively. It is concluded that when the questions are similar, the students’ scores in pharmacokinetic assessments are higher (medium effect size) in a remote open-book format compared with the in-person closed-book format.

## 1. Introduction

Open-book assessments in health sciences education have been studied for years before the pandemic [1,2,3,4,5,6,7,8,9,10,11,12,13,14]. With the exponential increase in the amount of knowledge in the medical and other health-related disciplines, like pharmacy, and the focus on the ability of the graduates to competently apply this knowledge, the use of open-book assessments seems to be inevitable [1,5]. It has been argued that with the technological advances in real-time access to knowledge, there is less need for clinical practitioners to rely exclusively on the memorization of facts during patient care [3]. Therefore, a more meaningful approach would focus on deep learning and higher cognitive functions and outcomes, which are more suited for open-book assessments.

Despite the potential benefits of open-book assessments, there is a lack of clarity in the literature regarding how the assessment format affects students’ performance in open- and closed-book examinations. A systematic review [11] of the literature in medical education concluded that although intuitively, it might be assumed that students would perform better in an open-book exam, most of the data in the literature show no significant differences between the two methods in examinee performance. The uncertainty about the potential differences between the two methods in terms of performance data might be due, at least in part, to different questions used in most studies for the two assessment formats. Indeed, in one study [6], where the students counter-intuitively performed better in the closed-book exam, the authors acknowledged that the open-book exam questions were more difficult. Additionally, students may spend less effort preparing for the open-book exam [2,14]. Therefore, even when the questions are identical in both assessment formats, the open-book format may not necessarily result in a higher score.

The assessment format became more crucial during the COVID-19 pandemic, which forced many higher education institutions to move to remote operations [15]. One of the main challenges facing educators during this transition was how to properly conduct summative assessments remotely [16,17]. Because of technological, financial, and, at times, emotional difficulties associated with monitoring students remotely during the assessments, open-book assessments became more attractive during the transition to online teaching. It has been suggested that the open-book format reduces the unfairness associated with some students violating academic integrity rules by consulting additional resources during the remote closed-book exams [17]. Therefore, many academic institutions used the open-book format for remote assessments during the COVID-19 pandemic [18,19], resulting in a renewed interest in comparing student performances in closed- and open-book assessments.

A recent study [18] in a Canadian pharmacy program reported that students generally performed better in remote open-book final examinations during the first year of the pandemic (2020) compared with a previous cohort (2019) who took the examinations in a closed-book in-person manner. However, the assumption of equivalency of the closed-book and open-book examinations could not be strictly controlled. The current study investigated the effects of exam format (in-person closed-book versus remote open-book) on students’ scores in different pharmacokinetic assessments containing identical questions for the two assessment formats. We hypothesized that the assessment format would not affect the scores of students.

## 2. Materials and Methods

### 2.1. Setting

Basic Pharmacokinetics is a three-credit-hour course offered during the second (Spring) trimester in an accelerated PharmD program. The format of the course [20] is based on the principles of active learning with substantial student engagement through online learning modules with opportunities for unlimited practice [21] and simulations [22]. Due to the COVID-19 pandemic in the Spring of 2020, the regularly scheduled 75-min, in-person sessions of the course were moved to synchronous remote format using the Zoom^®^ platform. The transition occurred during the 10th week of a 15-week trimester, consisting of 14 weeks of instruction and one week of final exams. However, except for the synchronous online delivery, the class format [20], including the required participation of students in the discussion during the class, remained unchanged. Additionally, all the in-person assessments that were closed-book were carried out remotely in an open-book format after the transition.

### 2.2. Design

**Figure 1:**
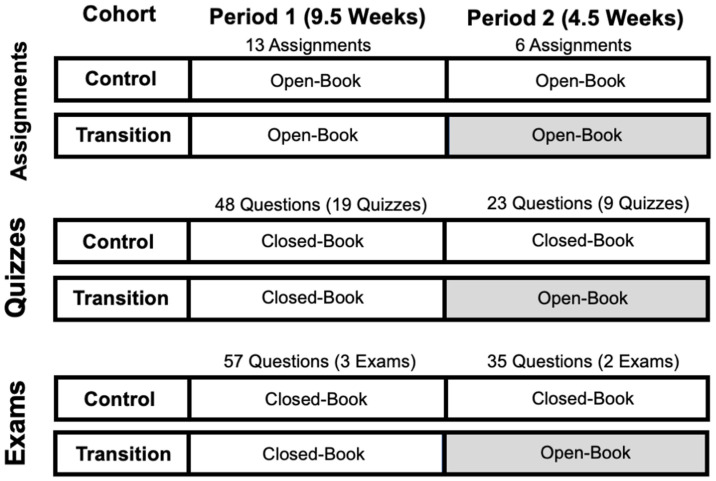
Graphic Representation of Study Design. The scores of two cohorts of students in pharmacokinetics Assignments, Quizzes, and Exams were compared during two periods (Period 1 and Period 2) in the trimester. Period 1 was in-person for both cohorts, whereas Period 2 was in-person for the Control cohort and remote for the Transition cohort. The shaded boxes indicate the remote period for the Transition cohort. The number of Assignments and Quiz and Exam questions in each period are also shown at the top of each period.

To analyze the effects of remote open-book assessment on students’ scores, the assessment data in the transition cohort (Transition, *n* = 96) were compared with the data for a prior cohort (Control, *n* = 85) for which the entire trimester was in person. Aside from the participation grade, the major assessments in the class were 19 take-home assignments, 28 daily online quizzes, and five exams (four progress and one final), which were included in the analysis, as demonstrated in Figure 1. Whereas the assessment format for the assignments before (Period 1) and after (Period 2) the transition to online teaching was the same (open-book), the daily quizzes and exams were closed-book before (Period 1) and open-book after (Period 2) the transition (Figure 1). However, all the other variables, such as the type of questions, the web-based method of administration, and the length of assessments for both the quizzes and exams, were the same for both periods.

To eliminate the effects of the difficulty of the quizzes and exam questions on the results, the Control cohort was selected to have the highest number of exam and quiz questions that matched the Transition cohort’s questions. There were 13 assignments, 48 quiz questions, and 57 exam questions in Period 1, which were identical in both cohorts and included in the analysis (Figure 1). For Period 2, the number of identical assessment items included in the analysis were six assignments, 23 quiz questions, and 35 exam questions (Figure 1). The exam questions were mapped to the three cognitive levels Foundational (remembering/understanding), Intermediate (applying), and Advanced (complex analyzing). Most of the exam questions (≥90%) were at the level of Intermediate and advanced. The individual students’ scores in each period’s quiz and exam categories were calculated by the percentage of correct answers to all the questions in each period, assuming identical weights for each question. The individual students’ scores in the assignment category were calculated by the average of all the assignment scores in each period, weighted by the number of questions in each assignment. The structure of the web-based, dynamic questions for the assignments [21] and quizzes/exams [23] have been described before. The modules create individualized questions for each student by incorporating random parameters in a question with an identical structure for all students but with different pharmacokinetic and/or dosing parameters. Therefore, although the questions are structurally the same, each student has unique, individualized questions.

### 2.3. Statitical Model

To analyze the data, separate linear mixed-effects regression models [24] were used for students’ assignments, quizzes, and exam scores. The fixed effects for the model were Cohort ($X_{Cohort}$, assigned 0 for Control and 1 for Test), Period ($X_{Period}$, assigned 0 for Period 1 and 1 for Period 2), and Cohort × Period Interaction ($X_{Cohort}\times X_{Period}$, assigned 1 for Test Cohort-Period 2 and 0 for others). The random effect was individual students’ intercept shift due to their academic differences from the group ($b_{i}$). The model is shown in the following equation:

$$Y_{ij}=\beta_{0}+X_{ijCohort}\beta_{Cohort}+X_{ijPeriod}\beta_{Period}+\left(X_{ijCohort}\times X_{ijPeriod}\right)\beta_{Cohort\times Period}+b_{i}+\in_{ij}$$

where $Y_{ij}$ is the score of the *i*^th^ student at the *j*^th^ measurement; $\beta_{0}$ is the population intercept; $\beta_{Cohort}$, $\beta_{Period}$, and $\beta_{Cohort\times Period}$ are the fixed effect coefficients for Cohort, Period, or Cohort × Period interaction, respectively; and $\in_{ij}$ refers to the unaccounted error. A Wald F test was used to perform significance tests on the fixed effects using Kenward and Roger’s method to adjust degrees of freedom, with pairwise comparisons for post-hoc analysis [25,26,27]. Cohen’s *d* effect sizes were then computed to show the magnitude of the differences [28]. The analysis used the R Project software for statistical computing version 3.6.2 [29]. The study was approved by our Institutional Review Board.

## 3. Results

**Figure 2:**
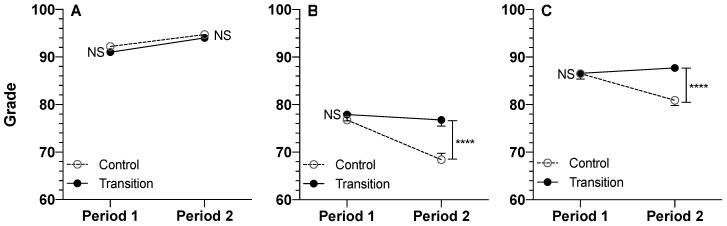
Grades of Students in the Control (In-Person) and Transition Cohorts in Assignments (**A**), Quizzes (**B**), and Examinations (**C**) for Period 1 and Period 2 in the Trimester. Mean (+ or − SE) values are presented for 85 Control and 96 Transition students. For Period 1 and Period 2, there were 13 and 6 Assignments, 48 and 23 Quiz questions, and 57 and 35 Exam questions, respectively, administered throughout the trimester. Whereas the Assignments were open-book in both periods, the Quizzes and Exams were open-book during Period 2 for the Transition cohort only. ****: *p* < 0.0001, post-hoc pairwise analysis of Control and Transition scores.

The students’ scores for the Transition and Control cohorts during the two periods for assignments, quizzes, and exams are depicted in Figure 2.

Additionally, the statistical analysis of the fixed effect parameters (Cohort, Period, and Cohort × Period interaction) and post-hoc analyses of the differences between the cohorts in their scores are presented in Table 1 and Table 2, respectively.

Analysis of the fixed-effect parameters with regard to the Cohort × Period interactions (Table 1) indicated there was no significant interaction for the assignments (*p* = 0.5833). In contrast, the interaction was significant (*p <* 0.0001) for both exams and quizzes. The significant Cohort × Period interactions for the quiz and exam questions indicate that the differences between the Cohorts in their quiz or exam questions (Table 1) are specific to a particular period (Period 2) (Figure 1).

Post-hoc data analysis (Table 2) indicated that for assignments administered in an open-book manner for both cohorts and periods, there were no significant differences between the two cohorts at either period (Figure 2). Additionally, for quizzes and exams, the scores for Period 1, when the format of the assessment was closed-book, were the same for both cohorts (Table 2, Figure 2). However, for Period 2, when the Transition cohort was switched to an open-book assessment format, the Transition cohort scored 8.42% (quizzes) or 6.82% (exams) higher (*p <* 0.0001) than the Control cohort, which continued to have closed-book assessments (Table 2, Figure 2). These increases in the scores because of the switch to an open-book format were equivalent to Cohen’s *d* effect sizes of 0.607 and 0.587 for the quizzes and exams, respectively (Table 1).

## 4. Discussion

The advantages and disadvantages of open-book assessments have been studied and debated by educators in the health sciences for many years, with the majority of studies carried out in medical schools [1,2,3,4,5,6,7,8,9,10,11,12,13,14]. It is generally believed that open-book assessments are better suited for questions that require higher cognitive levels rather than questions dealing with rote memorization [3,4,6,13,14]. Additionally, it has been argued that open-book assessments simulate the real-world environment better because practitioners have access to facts and data [3,11,13]. On the other hand, it has been reported that students may be spending less time preparing for open-book assessments [2,14], and access to resources may make them easy [13]. However, the current literature is ambiguous regarding students’ relative performance in the open- and closed-book assessments [11], which may be due to uncontrolled variables, such as different questions used in the two assessment formats. In the current study, we tested the effects of the open-book format on students’ performance using a design that controlled for several variables (Figure 1). Our results (Figure 2, Table 1 and Table 2) clearly show that when the questions in the two assessment formats are the same, the students score significantly higher in the remote open-book format. These results agree with a recent study [18] from a pharmacy program, which also showed higher exam grades for open-book assessments.

Regarding relevant literature in pharmacy education, a pre-pandemic study [4] addressed the performance and perceptions of students in open-book and closed-book assessments. That study used two groups of pharmacy students (Year 1 and Year 2) in a 4-year Bachelor of Pharmacy program to compare students’ scores in closed-book and open-book exams in a crossover design. Additionally, the study participation was voluntary; each assessment only had eight questions; different questions were used in the closed-book and open-book assessments; and the students were told that their performances would not affect their course grades. The authors reported the scores of students in the closed-book and open-book assessments after combining the data for the two groups of students (Year 1 and Year 2), which showed the open-book assessment had a higher score with a medium effect size (Cohen’s *d* effect size of 0.36). However, the data for comparisons of scores between the open-book and closed-book assessments were not reported separately for each group (Year 1 and Year 2). This may have been because of the test’s potentially low power in each group due to the small number of items (8 questions) in the assessments. Additionally, although the confounding effects of using different questions in the two assessment formats might have been mitigated by the crossover design of the study, the voluntary participation and formative nature of the assessments (no effect on the course grade) might limit the application of these findings to mandatory summative assessments. Nevertheless, the overall conclusion of that study [4] is in agreement with the results obtained in our current study, although we found a larger effect size (~0.6 versus 0.36).

A second study [18], which was reported recently, evaluated the effects of open-book final exams during the first year of the COVID pandemic (2020) on the performance of pharmacy students in a Canadian University. The authors compared students’ performance in 2020 with a control cohort 2019 in midterm (closed-book for both years) and final (open-book for 2020 and closed-book for 2019) exams. They reported that the ratios of the final exam over midterm exam scores during the pandemic year (2020) were higher than those for the control group (2019) for five of the seven courses evaluated, suggesting easier final exams during the open-book assessment in 2020. The results of our studies (Figure 2) agree with the finding of this report.

The magnitude of the differences between the scores in the open- and closed-book assessments (i.e., the effect size) was medium in our study, with scores in the open-book quizzes and exams being 8.42% and 6.82%, respectively, higher than those in the closed-book assessments (Table 2). However, the differences between the scores in the two assessment formats are likely affected by the cognitive level of the questions, with potentially larger differences for foundational knowledge-based questions whose answers can be easily found in an open-book format. At our school, faculty map their exam questions to one of the three cognitive levels Foundational (remembering/understanding), Intermediate (applying), and Advanced (complex analyzing). Our exams’ 35 questions in Period 2 (Figure 1) contained 3 Foundational, 24 Intermediate, and 8 Advanced questions. It is, however, possible that a different distribution of questions in terms of cognitive levels would have produced a different magnitude of difference. Additionally, the magnitude of the effect of open-book assessment on the scores may also be different for disciplines other than pharmacokinetics. Future studies are needed to test these postulates.

In addition to a significant Cohort × Period interaction for the quizzes and exams (Table 1), the statistical analysis of the fixed effect parameters also indicated a significant effect for the Cohort (*p <* 0.01) for these two assessments (Table 1). However, this significance is due to the Cohort × Period interaction, which resulted in significantly higher scores for the Transition cohort in Period 2 (Table 2, Figure 2). Indeed, the lack of significant differences between the two cohorts in their quiz and exam scores in Period 1 and their assignment scores in both periods (Figure 2 and Table 2) suggests no real differences between the academic capabilities of the two cohorts.

The data in Table 1 also indicate statistically significant effects for the Period for all three assessments. For the assignments, the significant Period effect in the absence of any Cohort × Period interaction is indeed due to more difficult assignments during Period 1 for both cohorts (Figure 2). However, the statistical significances of the Period effect for the quiz and exam questions (Table 1) primarily reflect the Cohort × Period interactions, resulting in students’ higher scores in Period 2 only (Figure 2 and Table 2).

It has been reported that by providing access to educational resources during the assessment, open-book assessments inherently reduce academic dishonesty [17,30]. This is especially important when costly and sometimes intrusive monitoring programs are not available or allowed during the remote assessments. For remote open-book assessments of individual students’ abilities, the potential for academic dishonesty mainly involves unwanted student collaborations during the assessment because they are allowed to use resources such as their notes or literature. Additionally, students may use recently available technological tools, such as generative artificial intelligence, to produce answers to the assessment questions. We instituted the following additional measures during our assessments to minimize the possibility of unwanted student collaboration or the use of advanced technological tools during the assessment: (a) use of individualized questions for each student, which requires different answers to the question for each student; (b) extensive use of questions that require analysis of data, including plotting and estimation of the pharmacokinetic parameters; (c) administration of the assessment at the same time to all students; and (d) keeping the length of the open- and closed-book assessments the same. We believe these measures have further reduced the chances of academic integrity violations during the remote open-book assessments.

Although our study examined the effects of open-book assessment in a remote setting, these assessments may also be administered in an in-person setting where they are proctored. In the in-person setting, the potential for academic integrity violations described above in a remote setting is eliminated or substantially minimized. Indeed, most pre-pandemic studies related to the open-book versus closed-book formats were performed in an in-person setting [1,4,5,6,9,12,14]. In their recent article [31], Dawson and his colleagues stated that the traditional closed-book versus open-book binary terms are inadequate for addressing the level of examination restrictions in the online and remote era. Indeed, there could be major differences between the in-person and remote open-book assessments.

Similar to the literature in the medical and pharmacy disciplines, the literature on the comparison of the open-book and closed-book assessments in other disciplines suggests that the open-book format is more suitable for assessments at higher cognitive levels [32,33]. Theophilides and Dionysiou [32] analyzed the open-book assessment literature and reported that the open-book exams do not result in higher test scores, particularly if the examinations require higher-order thinking. However, they reduce anxiety and stress, promote fair examination and long-term retention of concepts, and allow students to engage in deeper learning methods instead of rote memorization. The authors’ study of 173 sophomore students in a teacher education program concluded that open-book exams result in the creative use of knowledge, course content mastery, student self-evaluation and feedback, reduction of examination stress, and self-regulation in course studying. Similarly, Block [33] studied students’ performance in an Introductory Statistics course using three formats: closed-book, open-book, and closed-book with student-generated notecards. The assessments emphasized understanding and analysis as opposed to rote memorization. The author reported that students’ performance in the three assessment formats was generally comparable. However, students reported more satisfaction with the open-book formats. Overall, these studies suggest that open-book assessments do not necessarily result in higher scores if designed to test higher levels of learning.

A limitation of our study is that in addition to the different assessment formats (open- or closed-book), the teaching format was also changed for the two cohorts during Period 2 (remote versus in-person). Therefore, it might be argued that the observed differences between the two assessment formats (Figure 2, Table 2) are influenced by the differences in the effectiveness of the in-person and remote teaching delivery formats. However, this is unlikely because the scores of the two cohorts in the assignment category, which were assessed in an open-book manner in both cohorts and periods, were the same (Figure 2), regardless of the method of delivery of instruction (Figure 1).

Another limitation of our study is that although it shows that the open-book assessment of the same questions results in higher scores, the magnitude of the effect of open-book assessment on the scores will likely depend on the cognitive levels of the questions. We hypothesize that the higher the level of cognition of questions in Bloom’s taxonomy [34], the lower the difference in the scores between the closed-book and open-book assessments. Future studies are needed to test this hypothesis.

Overall, the results presented here agree with the previous reports [3,4,6,13,14] that the use of open-book assessments may be more suited for assessments at higher cognitive levels. For such assessments, an open-book format may also be used for in-person assessments.

## 5. Conclusions

Compared with a cohort with in-person closed-book assessments, students’ scores in pharmacokinetics quizzes and examinations were significantly higher in the cohort that received the assessments in a remote open-book format. Further studies are needed to determine the effects of the degree of difficulty of the assessment on the magnitude of the differences between students’ scores in the two assessment formats and the potential of our findings for extrapolation to other disciplines in pharmacy curricula.

## Figures and Tables

**Table 1:** Model-predicted significance of the fixed-effect parameters for assignments, quizzes, and exams.

Parameter	Cohort	Period	Cohort× Period Interaction
Assignment	0.2428	<0.0001	0.5833
Quiz	<0.01	<0.0001	<0.0001
Exam	<0.01	<0.05	<0.0001

**Table 2:** Post-Hoc, pairwise analysis of the Cohort × Period interactions for the differences between the Cohorts (Transition–Control).

Assessment	Period	Difference (Transition–Control)	SE	95% Confidence Interval	*p* Value	Cohen’s *d* Effect Size
Assignments	1	–1.20	0.938	−3.318, 0.907	0.19999	–
Assignments	2	−0.657	0.938	−2.77, 1.46	0.4844	–
Quizzes	1	+1.08	1.91	−3.22, 5.37	0.5735	–
Quizzes	2	+8.42	1.91	4.13, 12.7	<0.0001	0.607
Exams	1	+0.0593	1.50	−3.32, 3.44	0.9685	–
Exams	2	+6.82	1.48	3.48, 10.2	<0.0001	0.587

## Data Availability

The data presented in this study are available on request from the corresponding author. The data are not publicly available due to privacy.
